# LncRNA papillary thyroid carcinoma susceptibility candidate 3 (PTCSC3) regulates the proliferation of human periodontal ligament stem cells and toll-like receptor 4 (TLR4) expression to improve periodontitis

**DOI:** 10.1186/s12903-019-0802-9

**Published:** 2019-06-13

**Authors:** Wei Liu, Yongyang Zheng, Ben Chen, Ting Ke, Zhuojin Shi

**Affiliations:** 1 Department of Stomatology, The second Affiliated Hospital, School of Medicine, Zhejiang University, Hangzhou City, Zhejiang Province 310009 People’s Republic of China; 2 School of Stomatology, Zhejiang Chinese Medical University, No.548 Binwen Road, Hangzhou City, Zhejiang Province 310053 People’s Republic of China

**Keywords:** Periodontitis, Toll-like receptor 4, lncRNA PTCSC3, Periodontal ligament stem cells

## Abstract

**Background:**

LncRNA PTCSC3 is a known tumor suppressor, while its roles in other human diseases are unclear.

**Methods:**

All participants were admitted by The second Affiliated Hospital, School of Medicine, Zhejiang University from January 2016 to January 2018. RT-qPCR, Vectors, cell transfection, In vitro cell proliferation assay and western blot were using to carry out the experiment.

**Results:**

In this study we found that PTCSC3 was downregulated, while toll-like receptor 4 (TLR4) was upregulated in periodontal ligament stem cells (PDLSCs) isolated from periodontitis affected teeth that in PDLSCs isolated from healthy teeth. Expression levels of PTCSC3 and TLR4 were only significantly and inversely correlated in PDLSCs isolated from periodontitis affected teeth but not in PDLSCs isolated from healthy teeth. PTCSC3 overexpression led to the downregulation of TLR4 in PDLSCs isolated from periodontitis affected teeth, while TLR4 overexpression failed to significantly affect PTCSC3. PTCSC3 overexpression also led to the inhibited proliferation of periodontitis-affected PDLSCs.

**Conclusions:**

Therefore, lncRNA PTCSC3 may regulate the proliferation of PDLSCs and TLR4 expression to improve periodontitis.

## Background

Periodontitis is group of inflammatory disorders developed from the sub gingival biofilm after pathogenic bacteria infection [1]. Periodontitis is accompanied by connective tissue breakdown and impaired immune response [2]. During the development of periodontitis, cytokine production in gingival epithelium is accelerated by bacterial infections, leading to local or even systemic inflammatory responses and loss of tooth [3, 4]. The occurrence of periodontitis is affected by multiple risk factors, such as obesity, aging, diabetes [5]. With the growth of aging population and increase incidence of obesity and diabetes, prevalence of periodontitis is predicted to be significantly increased in near future [6]. Therefore, novel therapeutic targets for periodontitis are always needed.

It has been well accepted that only less than 2% of the transcriptions of mammalian genome is related to protein synthesis [7], most mammalian genes are only transcribed to non-coding RNAs (ncRNAs) [7], which lacks protein coding capacity but are critical determinants in many cellular processes [8]. A major subgroup of ncRNAs is long ncRNAs, which are composed of more than 200 nucleotides [9]. However, the function of most lncRNAs is still unknown and most lncRNAs studies are focused on certain lethal diseases, such as different types of cancer [10]. Studies in past several years also showed that the development of periodontitis is also accompanied by changed in expression patterns of a large set of lncRNAs [11]. Certain lncRNA, such ANCR and MEG3 regulate osteogenic differentiation to participate in the pathogenesis of periodontitis [12, 13]. LncRNA PTCSC3 has been characterized as a tumor suppressor [14], while its roles in other human diseases are unclear. Our RNA-seq data showed that PTCSC3 was likely downregulated in periodontitis, and its expression levels were inversely correlated with toll-like receptor 4 (TLR4), which promotes periodontitis [15]. Therefore, it will be reasonable to hypothesize that PTCSC3 may also participate in periodontitis. This study aimed to investigate the role of PTCSC3 in periodontitis and explored its interactions with TLR4 by performing overexpression experiments, which recovered the downregulation of PTCSC3.

## Methods

### Patients and PDLSCs

Periodontal ligament tissues were collected from periodontitis-affected third molars of 34 patients with periodontitis (patient group) during tooth extraction and form healthy third molars of 34 non-periodontitis patients (control group) who received orthodontic treatment. All participants were admitted by The second Affiliated Hospital, School of Medicine, Zhejiang University from January 2016 to January 2018. Patients complicated with other clinical disorders were excluded. According to the methods used by Zheng et al. [16], PDLSCs were isolated from periodontal ligament tissues and cultured. PDLSCs were collected from passage 5 to 7 for subsequent experiments. Patient group included 19 males and 15 females, and age ranged from 33 to 41 years, with a mean of 35.2 ± 3.1 years. Control group included 20 males and 14 females, and age ranged from 32 to 40 years, with a mean of 34.9 ± 3.5 years. This study has been approved by Ethics Committee of The second Affiliated Hospital, School of Medicine, Zhejiang University. All participants were informed the experimental principle and signed informed consent.

### Total RNA extraction and RT-qPCR

RNAzol reagent (Sigma-Aldrich, St. Louis, MO, USA) was directly mixed with in vitro cultivated PDLSCs to isolate total RNAs. SuperScript III First-Strand Synthesis System (Thermo Fisher Scientific) was used to synthesize cDNA through reverse transcription. To detect the expression of TLR4 and PTCSC3, SYBR™ Green PCR Master Mix (Cat: 4309155, Thermo Fisher Scientific) was used to prepare PCR reaction systems with GAPDH as endogenous control. Expression of TLR4 and PTCSC3 was normalized to GAPDH based on 2^-∆∆CT^ method. Expression levels of TLR4 and PTCSC3 in all samples were normalized to the sample with the lowest expression level, which was set to “1”.

### Vectors and cell transfection

PTCSC3 genomic DNA and TLR4 cDNA were inserted into pcDNA3.1 vectors to construct PTCSC3 and TLR4 expression vectors. The vector construction service was provided by Sangon (Shanghai, China). Lipofectamine 2000 reagent (Invitrogen, Carlsbad, USA) was used to transfect 10 nM vectors into 3 × 10^5^ PDLSCs in strict accordance with manufacturer’s instructions. For subsequent experiments, PDLSCs without transfections and PDLSCs transfected with empty vectors were used as control and negative control cells, respectively. PDLSCs were harvested at 36 h after transfected and overexpression of PTCSC3 and TLR4 were confirmed by RT-qPCR before subsequent experiments.

### In vitro cell proliferation assay

The proliferation ability of PDLSCs was tested at 36 h after transfection using Cell Counting Kit-8 (Dojindo Molecular Technologies, Inc.). Briefly, single cell suspensions were prepared and cell concentration was 3 × 10^4^ cells per ml. Cells were cultivated at 37 °C with 5% CO_2_ in a 96-well plate (100 μl cell per well). 10 μl CCK-8 solution was added into each every 2 h before the end of cell culture. After the termination of cell culture, 10 μl DMSO was added into each well and OD values were measured at 450 nm.

### Western blot

Total protein extraction kit (NBP2–37853, Novus Biologicals) was used to extract total protein from in vitro cultivated PDLSCs. Protein concentration was determined using BCA Protein Assay Kit (ab102536, Abcam). Following protein denature in boiled water for 5 min, electrophoresis was performed using 10% SDS denature PAGE gel with 30 μg per lane. Following gel transfer to PVDF membrane, blocking was performed in non-fat milk at room temperature for 2 h. After that, membranes were incubated with rabbit anti-human TLR4 (ab13867, 1:1200, Abcam) and GAPDH (ab9485, 1:1200, Abcam) at 4 °C overnight, followed by incubation with IgG-HRP (goat anti-rabbit, 1:1200, MBS435036, MyBioSource) for 2 h at room temperature. Signals were developed using Amersham ECL Western Blotting Detection Reagent (GE Healthcare). All data were processed using Image J v1.46 software.

### Statistical analysis

Three biological repeats were performed for each experiment and mean values were calculated for all data comparisons. Differences between patient and control groups were explored by unpaired t test. Difference among multiple cell groups were analyzed using ANOVA (one-way) and Tukey test. Correlations between PTCSC3 and TLR4 were analyzed by linear regression. Differences were statistically significantly when *p* < 0.05.

## Results

### PTCSC3 and TLR4 were dysregulated in periodontitis-affected PDLSCs

**Fig. 1:**
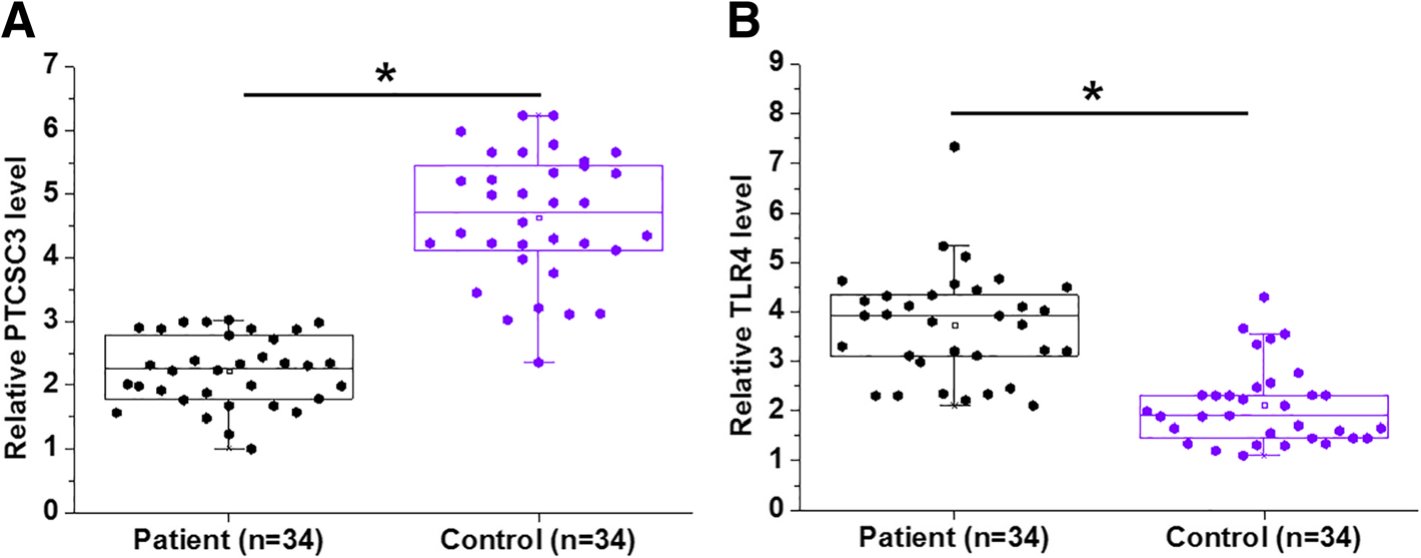
PTCSC3 and TLR4 were dysregulated in periodontitis-affected PDLSCs. RT-qPCR results showed that PTCSC3 was significantly downregulated (**a**), while TLR4 mRNA was significantly upregulated (**b**) in periodontitis-affected PDLSCs compared with healthy PDLSCs (*, *p* < 0.05)

Expression of PTCSC3 and TLR4 mRNA in 34 cases of periodontitis-affected PDLSCs and 34 cases of healthy PDLSCs was detected by RT-qPCR. Comparing to healthy PDLSCs, PTCSC3 was significantly downregulated (Fig. 1a), while TLR4 mRNA was significantly upregulated (Fig. 1b) in periodontitis-affected PDLSCs (*p* < 0.05), indicating the involvement of PTCSC3 and TLR4 in the pathogenesis of periodontitis.

### Expression levels of PTCSC3 and TLR4 were inversely correlated in periodontitis-affected PDLSCs

**Fig. 2:**
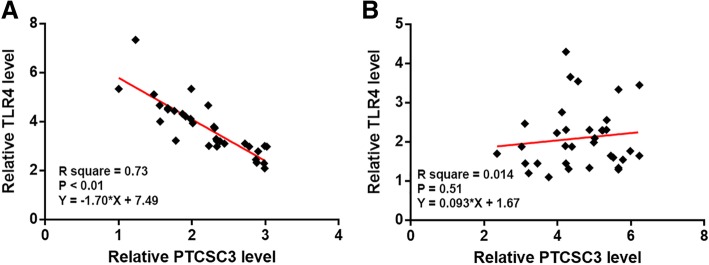
Expression levels of PTCSC3 and TLR4 were inversely correlated in periodontitis-affected PDLSCs. Linear regression showed that expression levels of PTCSC3 and TLR4 were significantly and inversely correlated in periodontitis-affect PDLSCs (**a**). In contrast, the correlation between PTCSC3 and TLR4 was not significant in healthy PDLSCs (**b**)

Linear regression showed that expression levels of PTCSC3 and TLR4 were significantly and inversely correlated in periodontitis-affect PDLSCs (Fig. 2a). In contrast, in healthy PDLSCs, the correlation between PTCSC3 and TLR4 was not significant (Fig. 2b).

### PTCSC3 mediated the downregulation of TLR4 in 2 cases of periodontitis-affected PDLSCs

**Fig. 3:**
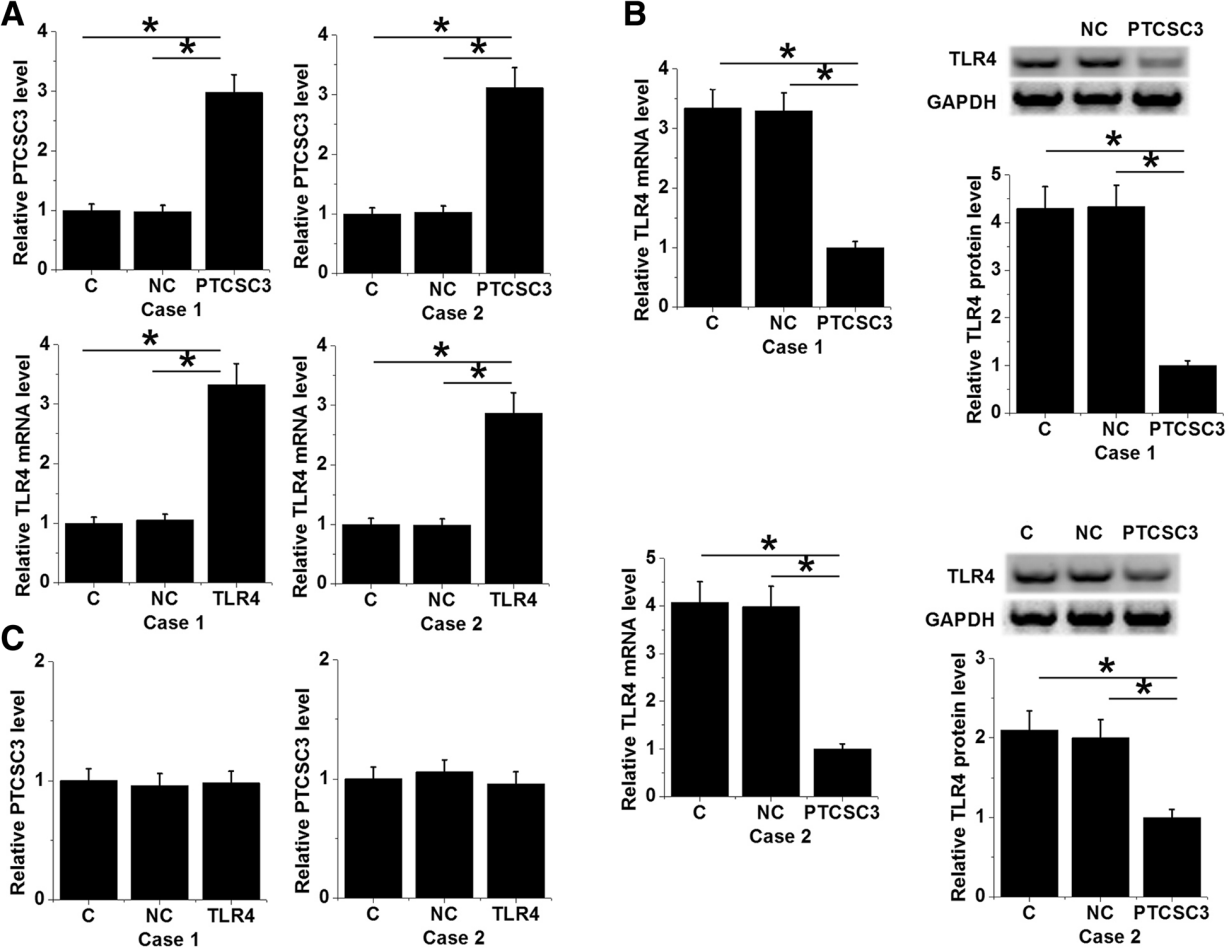
PTCSC3 mediated the downregulation of TLR4 in 2 cases of periodontitis-affected PDLSCs. Overexpression of PTCSC3 and TLR4 were confirmed by RT-qPCR at 36 h after transfection (**a**). PTCSC3 overexpression led to the downregulation of TLR4 in PDLSCs at both protein and mRNA levels (**b**), while TLR4 overexpression failed to significantly affect PTCSC3 (**c**), (*, *p* < 0.05)

Due the fact that PTCSC3 was downregulated in periodontitis, to recover this downregulation, this study performed PTCSC3 overexpression. To further investigate the interactions between PTCSC3 and TLR4 in periodontitis, PTCSC3 and TLR4 expression vectors were transfected into 2 cases of periodontitis-affected PDLSCs. Overexpression of PTCSC3 and TLR4 were confirmed by RT-qPCR at 36 h after transfection (Fig. 3a, *p* < 0.05). Compared with control (C) and negative control (NC) groups, PTCSC3 overexpression led to the downregulation of TLR4 in PDLSCs at both protein and mRNA levels (Fig. 3b, *p* < 0.05), while TLR4 overexpression failed to significantly affect PTCSC3 (Fig. 3c).

### Overexpression of PTCSC3 but not TLR4 led to the inhibited proliferation of periodontitis-affected PDLSCs

**Fig. 4:**
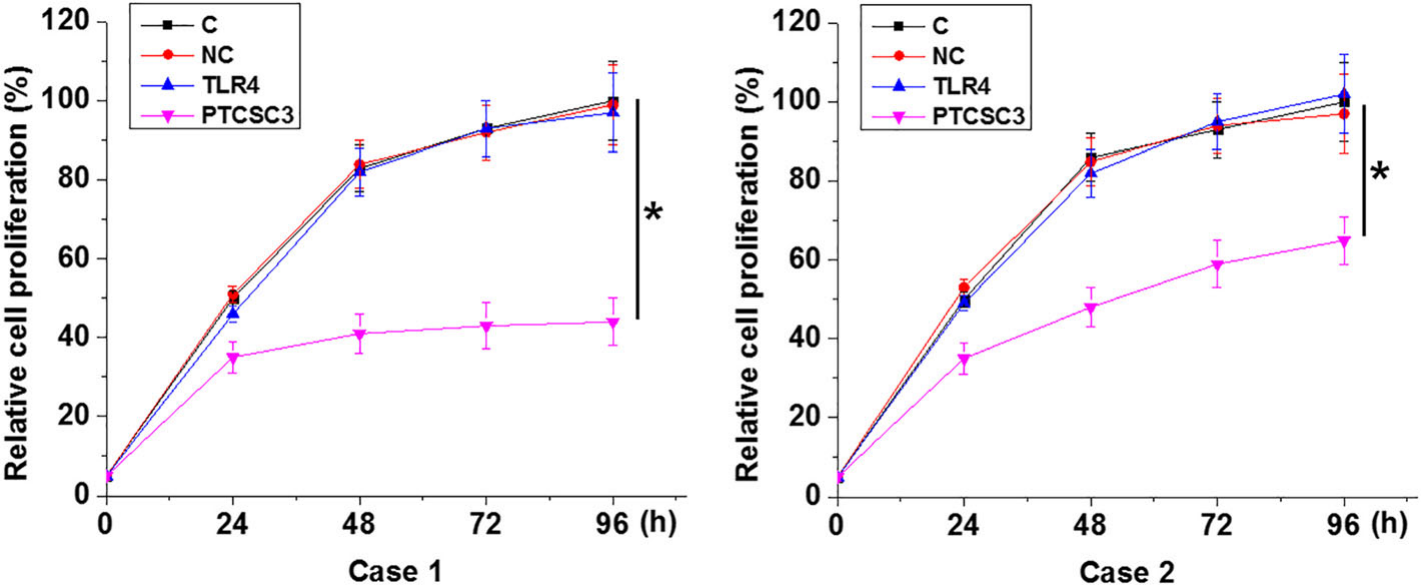
Overexpression of PTCSC3 but not TLR4 led to the inhibited proliferation of periodontitis-affected PDLSCs. PTCSC3 overexpression led to significantly inhibited proliferation of 2 cases of periodontitis-affected PDLSCs (*, *p* < 0.05). However, TLR4 failed to significantly affect the proliferation of periodontitis-affected PDLSCs

In vitro cell proliferation assay was performed to investigate the effects of PTCSC3 and TLR4 on proliferation of periodontitis-affected PDLSCs. Compared with control (C) and negative control (NC) groups, PTCSC3 overexpression led to significantly inhibited proliferation of 2 cases of periodontitis-affected PDLSCs (*p* < 0.05). However, TLR4 failed to significantly affect the proliferation of periodontitis-affected PDLSCs (Fig. 4).

## Discussion

LncRNAs are critical determinants in human diseases, while the function of lncRNAs in periodontitis is largely unknown. The key finding of the present study is that PTCSC3 was downregulated in periodontitis-affected PDLSCs and overexpression of PTCSC3 may improve periodontitis by inhibiting the proliferation of PDLSCs and downregulating the expression of TLR4.

TLR4 is a transmembrane protein that mediates the production of inflammatory cytokines involved in the activation of the innate immune system [17]. TLR4 is usually highly expressed in periodontal ligament mesenchymal stem cells and activation of TLR4 can mediate immune recognition of periodontal pathogens [18, 19], indicating its involvement in periodontitis. In a recent study, Ilievski et al. reported that TLR4 overexpression was closely correlated with insulin resistance and glucose intolerance in periodontitis [15]. Consistent with previous studies, our study also showed the upregulation of TLR4 in periodontitis-affected PDLSCs. The abnormal proliferation of PDLSCs contributes to the development of periodontitis [16]. In the present study, TLR4 overexpression showed no significant effects on proliferation of periodontitis-affected PDLSCs. Therefore, TLR4 may participate in periodontitis through PDLSCs proliferation-independent pathways.

PTCSC3 is a tumor suppressor in both thyroid carcinoma and glioma [20, 21], and overexpression of PTCSC3 inhibits tumor progression by inhibiting cancer cell proliferation. Consistently, our study also showed that PTCSC3 overexpression is an inhibitor of the proliferation of periodontitis-affected PDLSCs. Therefore, overexpression of PTCSC3 may serve as a potential therapeutic target for periodontitis. However, more clinical and experimental experiments are needed to further confirm this conclusion.

It is known that the expression of TLR4 can be regulated by certain lncRNAs [22]. Interestingly, our study proved that PTCSC3 was likely an upstream inhibitor of TLR4 in periodontitis-affected PDLSCs. Therefore, PTCSC3 may indirectly regulate immune recognition of periodontal pathogens by downregulating TLR4. It is worth noting that certain pathological mediators may exist between PTCSC3 and TLR4 due to the fact that expression levels of PTCSC3 and TLR4 were only significantly correlated in periodontitis-affected PDLSCs, but not in healthy PDLSCs. It is known that lncRNAs can sponge miRNAs to regulate their downstream targets [23]. Our future study will explore the possible involvement of miRNAs in the interaction between PTCSC3 and TLR4. However, a miRNA sponge usually upregulate its targets. In this study PTCSC3 overexpression led to downregulated not upregulated TLR4. Therefore, other mechanisms, such as certain signaling pathways involved in this interaction should also be examed.

## Conclusions

In conclusion, PTCSC3 was downregulated in periodontitis, and overexpression of PTCSC3 may improve periodontitis by inhibiting the proliferation of PDLSCs and downregulating TLR4.

## Data Availability

The datasets used and/or analyzed during the current study are available from the corresponding author on reasonable request.

## Abbreviations

ncRNAs: Non-coding RNAs
PDLSCs: Periodontal ligament stem cells
TLR4: Toll-like receptor 4

## References

[1] Meyle J, Chapple I (2015). Molecular aspects of the pathogenesis of periodontitis. Periodontol 2000.

[2] Hajishengallis G (2015). Periodontitis: from microbial immune subversion to systemic inflammation. Nat Rev Immunol.

[3] Silva N, Abusleme L, Bravo D, Dutzan N, Garcia-Sesnich J, Vernal R (2015). Host response mechanisms in periodontal diseases. J Appl Oral Sci.

[4] Sexton WM, Lin Y, Kryscio RJ, Dawson DR, Ebersole JL, Miller CS (2011). Salivary biomarkers of periodontal disease in response to treatment. J Clin Periodontol.

[5] Genco RJ, Borgnakke WS (2013). Risk factors for periodontal disease. Periodontol 2000.

[6] Nascimento GG, Leite FR, Do LG, Peres KG, Correa MB, Demarco FF (2015). Is weight gain associated with the incidence of periodontitis? A systematic review and meta-analysis. J Clin Periodontol.

[7] Consortium EP, Birney E, Stamatoyannopoulos JA, Dutta A, Guigo R, Gingeras TR (2007). Identification and analysis of functional elements in 1% of the human genome by the ENCODE pilot project. Nature..

[8] Mattick JS (2001). Non-coding RNAs: the architects of eukaryotic complexity. EMBO Rep.

[9] Furuno M, Pang KC, Ninomiya N, Fukuda S, Frith MC, Bult C (2006). Clusters of internally primed transcripts reveal novel long noncoding RNAs. PLoS Genet.

[10] Yan X, Hu Z, Feng Y, Hu X, Yuan J, Zhao SD (2015). Comprehensive genomic characterization of long non-coding RNAs across human cancers. Cancer Cell.

[11] Zou Y, Li C, Shu F, Tian Z, Xu W, Xu H (2015). lncRNA expression signatures in periodontitis revealed by microarray: the potential role of lncRNAs in periodontitis pathogenesis. J Cell Biochem.

[12] Jia Q, Jiang W, Ni L (2015). Down-regulated non-coding RNA (lncRNA-ANCR) promotes osteogenic differentiation of periodontal ligament stem cells. Arch Oral Biol.

[13] Liu Y, Zeng X, Miao J, Liu C, Wei F, Liu D (2019). Upregulation of long noncoding RNA MEG3 inhibits the osteogenic differentiation of periodontal ligament cells. J Cell Physiol.

[14] Wang XM, Liu Y, Fan YX, Liu Z, Yuan QL, Jia M (2018). LncRNA PTCSC3 affects drug resistance of anaplastic thyroid cancer through STAT3/INO80 pathway. Cancer Biol Ther.

[15] Ilievski V, Cho Y, Katwala P, Rodriguez H, Tulowiecka M, Kurian D (2015). TLR4 expression by liver resident cells mediates the development of glucose intolerance and insulin resistance in experimental periodontitis. PLoS One.

[16] Zheng W, Wang S, Wang J, Jin F (2015). Periodontitis promotes the proliferation and suppresses the differentiation potential of human periodontal ligament stem cells. Int J Mol Med.

[17] Vaure C, Liu Y (2014). A comparative review of toll-like receptor 4 expression and functionality in different animal species. Front Immunol.

[18] Marchesan J, Jiao Y, Schaff RA, Hao J, Morelli T, Kinney JS (2016). TLR4, NOD1 and NOD2 mediate immune recognition of putative newly identified periodontal pathogens. Mol Oral Microbiol.

[19] Trubiani O, Guarnieri S, Diomede F, Mariggio MA, Merciaro I, Morabito C (2016). Nuclear translocation of PKCalpha isoenzyme is involved in neurogenic commitment of human neural crest-derived periodontal ligament stem cells. Cell Signal.

[20] Wang X, Lu X, Geng Z, Yang G, Shi Y (2017). LncRNA PTCSC3/miR-574-5p governs cell proliferation and migration of papillary thyroid carcinoma via Wnt/beta-catenin signaling. J Cell Biochem.

[21] Xia S, Ji R, Zhan W (2017). Long noncoding RNA papillary thyroid carcinoma susceptibility candidate 3 (PTCSC3) inhibits proliferation and invasion of glioma cells by suppressing the Wnt/beta-catenin signaling pathway. BMC Neurol.

[22] Jin LW, Pan M, Ye HY, Zheng Y, Chen Y, Huang WW, et al. Down-regulation of the long non-coding RNA XIST ameliorates podocyte apoptosis in membranous nephropathy via the miR-217-TLR4 pathway. Exp Physiol. 2018;104(2):220-30. 10.1113/EP087190 30414341

[23] Bayoumi AS, Sayed A, Broskova Z, Teoh JP, Wilson J, Su H (2016). Crosstalk between long noncoding RNAs and MicroRNAs in health and disease. Int J Mol Sci.

